# Latent classes of posttraumatic stress disorder among survivors of the Bam Earthquake after 17 years

**DOI:** 10.1186/s12888-022-04216-3

**Published:** 2022-09-10

**Authors:** Elham Abolhadi, Parisa Divsalar, Mohammad Amin Mosleh-Shirazi, Tania Dehesh

**Affiliations:** 1 Department of Biostatistics and Epidemiology, School of Public Health, Kerman University of Medical Sciences, Kerman, Iran; 2 Neuroscience Research Center, Institute of Neuropharmacology, Department of Psychiatry, School of Medicine, Kerman University of Medical Sciences, Kerman, Iran; 3 Ionizing and Non-Ionizing Radiation Protection Research Center (INIRPRC), School of Paramedical Sciences, Shiraz University of Medical Sciences, Shiraz, Iran; 4 Physics Unit, Department of Radio-Oncology, Shiraz University of Medical Sciences, Shiraz, Iran; 5 Modeling in Health Research Center, Institute for Futures Studies in Health, Kerman University of Medical Sciences, Kerman, Iran

**Keywords:** Bam, Earthquake, Post-traumatic stress disorder, Risk factors, Latent class analysis

## Abstract

**Background:**

The purpose of this study was to identify latent classes of the severity of post-traumatic stress disorder (PTSD) among the survivors of the earthquake in Bam, south-eastern Iran, 17 years after the disaster. The most influential predictor variables of PTSD classes were also investigated.

**Methods:**

Eight hundred survivors of the Bam earthquake who were at least four years old in that disaster were selected by multistage sampling. The PTSD Checklist-Civilian Version was used. Latent class analysis was performed to identify subgroups of people with different PTSD symptom profiles. Latent class regression analysis was used to explore the influence of demographic and traumatic variables on each class membership.

**Results:**

We found three latent classes of PTSD, with the following profiles emerging: Low Symptom (56.6% of the participants), Moderate Symptom (23.5%), and Severe Symptom (19.9%). Old age [OR = 2.20, 95% CI = (1.46, 3.32)], physical injury [OR = 1.95, 95% CI = (1.24, 3.06)], being trapped under the rubble [OR = 1.81, 95% CI = (1.15, 2.86)], and death of a family member [OR = 1.86, 95% CI = (1.12, 3.09)] were positive risk factors for PTSD and increased the chance of being in classes having more severe symptoms. Having a high educational level was a negative risk factor [OR = 0.86, 95% CI = (0.67, 1.11)].

**Conclusions:**

The severity of PTSD 17 years after the earthquake shows that natural disasters such as earthquakes have long-term consequences, and earthquake survivors must have psychological support and long term health care.

After any catastrophic earthquake, governments should establish psychology and psychotherapy centers for earthquake victims, and these centers should support earthquake victims for a sufficiently long time.

## Background

The Bam earthquake was one of the most devastating earthquakes in Iran [1]. Measuring 6.3 on the Richter scale, it struck the city of Bam in Kerman Province, south-eastern Iran, for 10 s on the 26th of December 2003 at 5.26 A.M. More than 40,000 people were killed and about 30,000 others were injured, nearly 20,000 homes were destroyed and more than 45,000 people were left homeless [2, 3].

The main structure of the buildings in Bam was made of mud brick. Bam Citadel is the largest brick building in the world, which is registered by UNESCO. The benefit of mud brick buildings was providing cool temperatures in summer and warmth in the winter in the house. mud brick is, however, not a strong building material and can suffer heavy damage in earthquakes.

Being injured, seeing dead bodies, loss of homes, communities, jobs, family members, friends, neighbors, and colleagues are important stressful life events that are usually experienced by survivors of a disaster such as the Bam earthquake. As a result, survivors may be at risk for stress-related disorders, such as post-traumatic stress disorder (PTSD) [4].

PTSD is one of the most common mental disorders caused by unexpected horrible events [5]. It is a type of disorder that can occur after exposure to exceptionally horrifying events such as war, sexual or physical abuse, a serious accident, or a natural disaster such as a fire, tornado, flood, or earthquake. This disorder is defined as experiencing three types of persistent symptoms following a traumatic event: re-experiencing, avoidance and increased arousal [6].

Natural disasters involving massive casualties, such as earthquakes, can lead to dangerous side effects that can last for several years. Hence, following up the mental disorder status of earthquake survivors is crucial, because in some cases, mental disorders such as PTSD can last for a very long time, perhaps for the remainder of the individual’s life. Moreover, most people with longstanding PTSD find that the symptoms are not steady in their severity. Consequently, people with these symptoms should not be forgotten, and it is vital for disaster mental health services to pay more attention to them, and systematically screen for this disorder.

One of the powerful data driven statistical methods is latent class analysis (LCA), which divides observations into different classes such that observations in each class are very similar to each other while observations from other classes are different [7]. This method has the potential to identify similar patterns of responses to questionnaire items and thereby identify hidden classes of observations [7, 8]. Thus, people with similar sets of responses to the questionnaires will tend to be grouped into the same latent classes. An extension of the LCA is the latent class regression (LCR) analysis, which allows the inclusion of various covariates to predict latent class membership with more precision [9, 10].

Latent class analysis has a series of advantages compared to conventional clustering techniques such as k-means clustering: first, selection of a cluster criterion is less arbitrary because of the underlying statistical model. Second, being a model-based approach, it provides several rigorous statistical tests to assess the model fit. Third, it also provides formal criteria to make decisions about the appropriate number of clusters. Fourth, latent class analysis can include observed variables of different scaling and measurement levels. Finally, the probabilistic nature of cluster membership in latent class analysis leads to less biased estimations of class-specific means, as each case only contributes to this mean weighted by its class membership probability [11].

Several studies have used LCA to extract best fitting classes of individuals with different PTSD symptom presentations. Maguen, S., et al. (2013) [12] examined latent classes of PTSD symptoms in Iraq and Afghanistan Veterans. Four latent classes of PTSD were identified. These classes were labeled High Symptom, Intermediate Symptom, Intermediate Symptom with Low Emotional Numbing, and Low Symptom with 34%, 41%, 10% and 15% of the participants, respectively. J. Rosellini, A.J., et al. (2014) [13] used LCA for a sample of participants who were affected by Hurricane Katrina. They found four latent classes of PTSD named Severe, Moderate, Mild, and Negligible Classes. Also, it was demonstrated that potentially traumatic hurricane-specific experiences, pre-hurricane traumatic events, co-occurring depression symptom severity and suicidal ideation, certain religious beliefs, and post-hurricane stressors were factors that influenced membership in the Severe and Moderate Classes. Eisma, M.C., et al. (2018) [4] conducted LCA to determine patterns of PTSD and Complicated Grief (CG) in a sample of bereaved Sichuan earthquake survivors one year after the disaster. That study reported five distinct PTSD and CG symptom: CG class with low PTSD and high CG, a combined class with high PTSD and high CG, a class with low PTSD and partial CG, a class with partial PTSD and CG, and a resilient class with low PTSD and CG. Furthermore, differences between the classes were examined according to seven demographic, disaster-related, and loss-related variables.

Understanding the presence and predictors of different PTSD classes may be particularly important in identifying factors that are associated with experiencing PTSD symptoms at varying levels of severity. To that end, the aim of present study was to explore unobserved classes of PTSD based on questionnaire responses in survivors of the Bam earthquake after 17 years. In addition, after identifying the optimal number of latent classes through LCA, we evaluated predictors of class membership by using an LCR analysis.

This study seeks to answer the hypothesis of whether PTSD exists after 17 years in Bam earthquake survivors. If so, how many latent PTSD classes can be found, and what are the main effective predictors of latent PTSD class membership.

## Methods

### Participants and settings

Eight hundred people who experienced the Bam earthquake participated in this cross-sectional study carried out in September 2020, approximately 17 years after the event. All people living in this city at the time of the earthquake aged 16 years or older at the time of the study (i.e., were at least 4 years old at the time of the earthquake) were enrolled in the study.

Multistage sampling was applied to obtain a representative sample of individuals living in Bam. The city of Bam was divided into five urban divisions and a number of houses were selected randomly from each part. Random sampling was based on a list of all households derived from the electricity bills. This research was carried out by a door-to-door approach (home visit).

The study inclusion criteria were: residing in Bam city at the time of the earthquake, being at least 4 years old in that time, and having no difficulty in understanding the questions or communicating. The exclusion criterion was: Individuals with mental disability, dementia or psychosis at the time of interview.

The purpose of the study was explained to all study participants, and their verbal informed consent was obtained before starting the interview and collecting information from the respondents. Demographic information including gender, age, marital status, education level, and traumatic characteristics about home loss, deaths of family, friends, relatives, and work colleagues, having limb-threatening injury and surgery, and being trapped under the rubble were collected. Participants' demographic information was collected using a checklist. Then, the 17-item PTSD Checklist–Civilian Version (PCL-C) was completed on all subjects by our trained team. The responses of illiterate and elderly people (people more than 60 years old) were confirmed by a witness.

Ethical approval for this study was granted by the Ethics Committee of the Kerman University of Medical Sciences)approval number 1400/177( and the verbal informed consent was approved by this Ethics Committee too. All procedures performed in this study were in accordance with the 1964 Helsinki Declaration and its later amendments.

Participants with missing information were not included in the analysis. Therefore, participants without complete data (*n* = 14, 1.75%) were excluded from the analysis.

### Instruments

PTSD symptoms were measured with the PTSD Checklist for the Diagnostic and Statistical Manual of Mental Disorders, Fourth Edition (DSM-IV). The PCL-C is a 17-item self-report measure reflecting DSM-IV symptoms of PTSD. It was developed by the Behavioral Science Branch of American PTSD Research Center in 1994 for evaluating the experience of ordinary people after trauma in normal life. Each item describes a symptom of PTSD, corresponding to the three DSM-IV symptom dimensions of PTSD. The first dimension is re-experiencing of the traumatic events (items 1–5); the second dimension is scheduled avoidance of trauma-relevant stimuli and numbing of general responsiveness (items 6–12), and the third dimension comprises symptoms of hyper-arousal (items 13–17). Participants were asked to indicate how much they have been bothered by each symptom in the past month using a 5-point (1–5) scale from 1 (Not at all) to 5 (Extremely) and a total symptom severity score (range = 17–85) was obtained by summing the scores from each of the 17 items [14]. Validity and reliability of the Persian version of the PTSD questionnaire has already been assessed in Iran; the Cronbach’s alpha coefficient scale was calculated at 0.93, which shows acceptable reliability in Persian [15].

### Statistical analyses

In order to explore latent classes for allocating people based on PTSD questionnaire responses, the LCA and latent class regression analysis were carried out.

#### Latent class analysis

LCA is a method for analysis of categorical data introduced by Lazarsfeld in (1950). It is a statistical technique which identifies unobserved classes of individuals based on patterns of their responses to the items of questionnaires. Thus, the aim of LCA is finding unobserved (latent) classes in order to classify the respondents to questionnaires [16]. In LCA construction for PTSD, for instance, items scored as 1 = ‘Not at all’ or 2 = ‘A little bit’ were coded as symptom absent, and items scored as 3 = ‘Moderately’, 4 = ‘Quite a bit’, or 5 = ‘Extremely’ were coded as symptom present.

The basic LCA relationship is given by

$$P\left(\uptheta \right)=\sum_{k=1}^K{\pi }_{k} {p}_{j}\left({\uptheta }_{j}\right)$$

where ${Y}_{i}$ is the $i$th observation of the manifest variables (the items of questionnaires), $K$ is the number of classes and ${\pi }_{k}$ is the initial probability of membership in class $k$ and ${p}_{j}$ is the class specific probability of ${Y}_{i}$ given the cluster specific parameters ${\uptheta }_{j}$.

Class estimation was carried out using the Expectation–Maximization (EM) algorithm [17]. The optimal number of latent classes for this LCA was selected based on the Bayesian Information Criterion (BIC) and Akaike information criterion (AIC). Theoretically, the best fitting LCA model has the lowest BIC and AIC [18, 19]. To determine the optimal number of classes, the LCA method was applied to the data for K = 2, …, 8 classes without predictor variables. BIC and AIC of these classes were generated and compared. The models were run starting with a two-class solution, and increasing the number of classes by one, until the BIC and AIC began to increase between consecutive models.

#### Latent class regression analysis

The LCR analysis is a generalized form of LCA that investigates the effect of independent variables on construction of LCA. In LCA, each observation has the same initial probability of latent class membership, whereas in the LCR analysis, the initial probabilities for each observation are different based on the effects of independent variables [18, 19]. After selecting the most optimal class solution, people are allocated to the classes with more precision by including these variables as covariates in the model.

The LCR analysis can be defined as

$$P\left(\uptheta \right)=\sum_{k=1}^K{\pi }_{k} \left({X}_{i};{\beta }_{k}\right){p}_{j}\left({\uptheta }_{j}\right).$$

where, ${X}_{i}$ represents the observed covariate for individual $i$ and ${\beta }_{k}$ denotes the vector of coefficients corresponding to the $k$ th latent class.

Descriptive statistics were generated for all variables using the Statistical Package for Social Science (SPSS) – version 25 for Windows. All other analyses were performed using R version 4.0.3. The LCA and LCR analysis were written using the poLCA function from the R-package poLCA.

## Results

The numbers of male and female participants were almost equal. the mean age was 44.52 years (standard deviation (SD) = 10.39 years). The majority of participants were single (*n* = 705, 89.7%) and the numbers of them who were married, or widowed were (*n* = 58, 7.4%), and (*n* = 20, 2.6%), respectively. Table 1 summarizes the demographic characteristics of participants and descriptive findings. It also presents the comparison of PTSD scores in different variable levels.

**Table 1:** Demographic characteristics of the participants

Variable	Levels	Number (Percent)	Re-experience	*p*-value	Avoidance	*p*-value	Hyperarousal	*p*-value	total	*p*-value
			**Mean ± S D**		**Mean ± S D**		**Mean ± S D**		**Mean ± S D**	
Age	-	786 (100%)	13.80 ± 3.55	-	17.90 ± 4.06	-	12.80 ± 3.45	-	44.52 ± 10.39	-
Gender										
	Male	396 (50.4%)	13.65 ± 3.51	0.248	17.80 ± 4.17	0.365	12.67 ± 3.43	0.276	44.09 ± 10.56	0.230
	Female	390 (49.6%)	13.98 ± 3.58		18.01 ± 3.95		12.98 ± 3.46		44.97 ± 10.21	
Education Level										
	Illiterate/ drop out	403 (51.3%)	14.31 ± 3.76	0.010	18.48 ± 4.07	0.001	13.39 ± 3.48	< 0.001	46.16 ± 10.57	< 0.001
	Graduate	264 (33.6%)	13.34 ± 3.30		17.24 ± 4.00		12.18 ± 3.34		42.78 ± 10.07	
	Associate Degree	47 (6.0%)	13.06 ± 2.94		16.90 ± 3.93		12.20 ± 3.12		42.16 ± 9.44	
	Bachelor Degree	55 (7%)	13.30 ± 3.20		17.96 ± 3.77		12.41 ± 3.39		43.69 ± 9.63	
	Postgraduate	7 (0.9%)	13.42 ± 3.95		17.28 ± 4.95		12.71 ± 4.15		43.42 ± 12.73	
Marital Status										
	Single	705 (89.7%)	13.90 ± 3.55	0.130	17.90 ± 4.03	0.139	12.90 ± 3.46	0.108	44.77 ± 10.37	0.035
	Married	58 (7.4%)	12.82 ± 3.45		17.08 ± 4.35		11.87 ± 3.25		41.79 ± 10.45	
	Widow	20 (2.6%)	15.78 ± 2.11		20.17 ± 2.71		14.24 ± 3.51		40.18 ± 6.01	

*p*-value < 0.05 is significant

The prognostic indices for the candidate models are shown in Table 2. The three-class solution was selected because its BIC and AIC were lower than the other class solutions.

**Table 2:** Latent class solutions and fit indices

Model	BIC	AIC	log-likelihood	ssBIC	VLMR	**VLMR –*P*-value**	
Class 1	32,175.40	32,241.40	-15,867.69	29,131.2	123.7	0.071	
Class 2	26,139.88	26,272.88	-12,626.59	23,982.4	92.4	0.421	
**Class 3**	**25,785.28**	**98,255.28**	-12,225.95	**23,774.2**	**83.2**	0.341	
Class 4	25,902.07	26,169.07	-12,060.99	23,789.9	83.4	0.323	

*BIC* Bayesian information criterion, *AIC* Akaike information criterion, *ssBIC* Sample-Size Adjusted BIC, *VLMR* Vuong-Lo-Mendell-Rubin

**Fig. 1:**
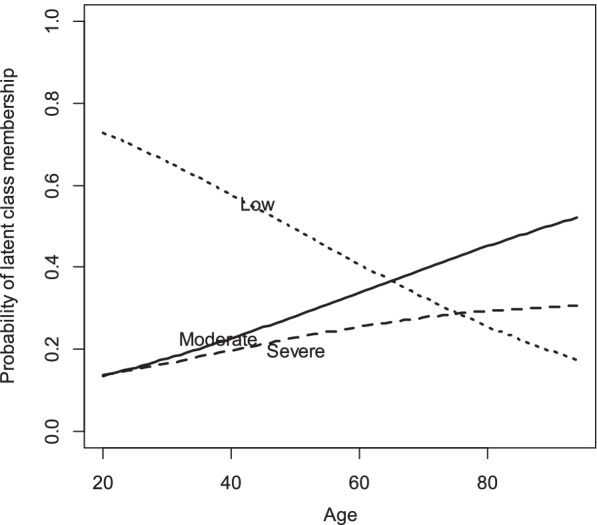
Predicted probabilities of latent class membership at varying levels of age

Table 3 shows interpretation and naming of each class based on the percentage of answers to the options of each item. The first class, which was the largest one (*n* = 445, 56.6%), was characterized by a very high probability of endorsing “A little bit” (code 2) for most of the 17 items of questionnaire. This class was, therefore, labelled the ‘Low class’. The second class (*n* = 185, 23.5%) was characterized by a high probability of endorsing “Moderately” (code 3) for each of the 17 items of the questionnaire. We labelled this the ‘Moderate class’. The third class (*n* = 156, 19.9%) was also highly likely to endorse “Moderately” (code 3) and “Quite a bit” (code 4). This class was, therefore, labelled the ‘Severe class’. Figure 1 shows that in each class the pattern of bars (labeled ‘S1’ through ‘S17’) are similar.

**Table 3:** Item response probabilities for each item in different latent classes

Question	Low class					Moderate class					Severe class				
	**Item options**					**Item options**					**Item options**				
	**1**	**2**	**3**	**4**	**5**	**1**	**2**	**3**	**4**	**5**	**1**	**2**	**3**	**4**	**5**
Question 1	0.03	**0.80**	0.17	0.00	0.00	0.00	0.30	**0.65**	0.05	0.00	0.00	0.09	**0.69**	0.19	0.03
Question 2	0.03	**0.61**	0.35	0.01	0.00	0.00	0.19	**0.69**	0.12	0.00	0.00	0.05	**0.53**	0.38	0.04
Question 3	0.08	**0.49**	0.42	0.01	0.00	0.00	0.15	**0.45**	0.40	0.00	0.00	0.04	0.31	**0.56**	0.09
Question 4	0.09	**0.52**	0.38	0.00	0.00	0.00	0.03	0.10	**0.79**	0.08	0.00	0.02	0.16	**0.54**	0.28
Question 5	0.13	**0.60**	0.27	0.00	0.00	0.01	0.06	**0.51**	0.42	0.00	0.00	0.01	0.34	**0.55**	0.10
Question 6	0.04	**0.85**	0.10	0.00	0.01	0.00	0.39	**0.61**	0.00	0.00	0.01	0.06	**0.89**	0.04	0.00
Question 7	0.06	**0.69**	0.25	0.00	-	0.00	0.31	**0.68**	0.01	-	0.01	0.08	**0.65**	0.26	-
Question 8	0.14	**0.46**	0.40	0.00	-	0.01	0.32	**0.61**	0.06	-	0.00	0.18	**0.48**	0.34	-
Question 9	0.15	**0.53**	0.32	0.00	0.00	0.00	0.24	**0.65**	0.10	0.01	0.00	0.05	0.41	**0.53**	0.01
Question 10	0.15	**0.55**	0.30	0.00	0.00	0.00	0.21	**0.61**	0.18	0.00	0.01	0.03	0.41	**0.52**	0.02
Question 11	0.14	**0.60**	0.26	0.00	0.00	0.00	0.18	**0.51**	0.31	0.00	0.00	0.03	0.39	**0.52**	0.06
Question 12	0.19	**0.55**	0.26	0.00	0.00	0.01	0.10	**0.54**	0.35	0.00	0.00	0.03	0.43	**0.49**	0.05
Question 13	0.13	**0.62**	0.25	0.00	0.00	0.00	0.05	0.26	**0.66**	0.03	0.00	0.02	0.35	**0.53**	0.10
Question 14	0.10	**0.63**	0.27	0.00	0.00	0.00	0.04	0.26	**0.69**	0.01	0.00	0.02	0.17	**0.66**	0.15
Question 15	0.18	**0.61**	0.21	0.00	0.00	0.00	0.07	**0.78**	0.14	0.01	0.00	0.02	**0.62**	0.35	0.01
Question 16	0.25	**0.58**	0.17	0.00	0.00	0.00	0.33	**0.66**	0.01	0.00	0.00	0.07	**0.77**	0.15	0.01
Question 17	0.18	**0.69**	0.13	0.00	0.00	0.01	0.41	**0.58**	0.00	0.00	0.00	0.30	**0.60**	0.08	0.02
**Probability of latent class membership**	**56.6%**					**23.5%**					**19.9%**				

Bolded items show the highest percentages of presence for each item’s option

1 = Not at all, 2 = A little bit, 3 = Moderately, 4 = Quite a bit, 5 = Extremely

Table 4 presents the latent class regression model results. The results of the model showed that among the descriptive variables age, deaths of family, friends, relatives, and work colleagues, having limb-threatening injury and surgery and being trapped under the rubble were the positive significant factors on class finding that increase the chance of membership in the moderate and severe classes. Also, having a high level of education was protective for PTSD and decreased the probability of membership in the moderate and severe classes.

**Table 4:** Odds Ratios and 95% Confidence Intervals from the latent class regression model

Participant Demographics	Moderate vs. Low		Severe vs. Low	
	**OR (CI 95%)**	***p*-value**	**OR (CI 95%)**	***p*-value**
Age	2.53 (1.58, 4.05)	< **0.001**	2.20 (1.46, 3.32)	< **0.001**
Gender				
women vs. men	1.61 (0.94, 2.77)	0.088	0.98 (0.62, 1.53)	0.932
Education level				
4 vs. 0	0.66 (0.52, 0.84)	**0.001**	0.86 (0.67, 1.11)	0.261
3 vs. 0	0.58 (0.47, 0.78)	**0.001**	0.74 (0.58, 1.01)	0.271
2 vs. 0	0.51 (0.49, 0.68)	**0.001**	0.62 (0.51, 1.06)	0.251
1 vs. 0	0.47 (0.41, 0.61)	**0.002**	0.58 (0.48, 1.03)	0.282
Marital status				
Single vs. Married	0.96 (0.66, 1.41)	0.867	1.03 (0.64, 1.57)	0.989
Widow vs. Married	0.89 (0.78, 1.11)	0.781	1.08 (0.58, 1.32)	0.872
Losing house				
Yes vs. No	3.64 (0.86, 15.28)	0.078	2.20 (0.60, 8.17)	0.242
Death of friend relatives and work colleagues				
Yes vs. No	1.98 (1.23, 3.18)	**0.005**	1.86 (1.12, 3.09)	**0.017**
Being injured and Having surgery				
Yes vs. No	2.02 (1.36, 2.99)	**0.001**	1.95 (1.24, 3.06)	**0.004**
Being trapped under the rubble				
Yes vs. No	2.11 (1.41, 3.16)	< **0.001**	1.81 (1.15, 2.86)	**0.010**

Bolded *p*-value = *p*-value < 0.05 is significant

OR Odds ratio, *CI* Confidence interval

0 = Illiterate/drop out, 1 = Graduate, 2 = Associate Degree, 3 = Bachelor Degree, 4 = Postgraduate

The Low class was the reference group and the other groups were compared with it. Compared to the Low class, people who were physically injured and trapped under the rubble were more than twice more likely of belonging to Moderate class, [OR = 2.02, 95% CI = (1.36, 2.99)] and [OR = 2.11, 95% CI = (1.41, 3.16)], respectively. Moreover, those who had lost a family member were nearly twice more likely to be in this class [OR = 1.98, 95% CI = (1.23, 3.18)]. Also, participants who were physically injured, trapped under the rubble and had lost a family member had more chance of membership in the Severe class [OR = 1.95, 95% CI = (1.24, 3.06)], [OR = 1.81, 95% CI = (1.15, 2.86)] and [OR = 1.86, 95% CI = (1.12, 3.09)], respectively. There was also a statistically significant education difference between Moderate and Low classes [OR = 0.66, 95% CI = (0.52, 0.84)], whereas no education difference was found for the Severe class compared to the Low class [OR = 0.86, 95% CI = (0.67, 1.11)].

**Fig. 2:**
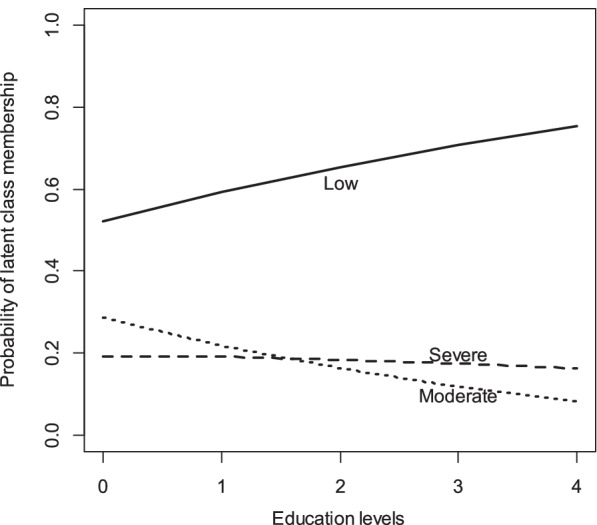
Predicted probabilities of latent class membership at varying levels of education

Figure 1 shows that with increase age, the probability of being in the low class decreased and, conversely, the probability of being in the moderate and severe classes increased. Figure 2 illustrates that by increasing the level of education, the probability of being in the low class rises, the probability of being in the Moderate class fell and the probability of being in the severe class remained stable.

## Discussion

The aim of the present study was to identify latent classes of PTSD and predictors of latent class membership in a sample of survivors of Bam earthquake 17 years later, using LCA and latent class regression.

The results of this study show that 17 years after the Bam earthquake, a number of survivors still suffer from PTSD. In the current study, participants were classified into three latent classes on the basis of severity and prevalence of PTSD symptoms. The largest PTSD class was individuals with the Low Symptom (56.6%) profiles and the two other classes were Moderate and Severe classes (23.5% and 19.9%, respectively). In addition, our study demonstrates that old age, physically injury, being trapped under the rubble, and deaths of a family member were risk factors of PTSD and high education level was a preventive risk factor of PTSD.

We found three latent classes for the severity of PTSD. This is in accordance with a study that conducted one year after the 2008 Wenchuan earthquake in China, where people were classified into three classes on the basis of severity and prevalence of PTSD symptoms [20]. According to that study, low symptoms class (10.3%) was the smallest one, while in the current study a majority of the individuals were in the Low class (56.6%). It may be due to the fact that this study was conducted 17 years after the earthquake as opposed to one year and, therefore, assesses the long-term psychological effects of trauma exposure. Our results in terms of prevalence of PTSD are in line with a research that was conducted 2.5 years after the Wenchuan earthquake, in which classes were characterized by varying severity and pervasiveness of PTSD and dysexecutive symptoms and the smallest class was the extremely high symptom one (3.7%) [21]. Comparing those results to this research makes it further clear that after many years, the prevalence of PTSD was still fairly high and these people still suffer from this disorder. It illustrates the importance of psychological treatments for the affected population.

This study indicates that older people were more likely to fall into the Moderate and Severe classes compared to the Low class. These findings generally agree with studies showing that being older was associated with increased risk of PTSD [22–24]. The link between older age and PTSD is probably related to loss of valuable belongings such as house, and persistent financial problems after the traumatic event. By contrast, one study carried out elsewhere by Kun, P., et al., [25] has demonstrated that age is not associated with increased risks for PTSD.

In the current study, positive significant relationships were found for being injured, being trapped under the rubble, and have lost a family member. They were associated with higher probability of being classified into the Moderate and Severe classes than compared with the Low class. Our study supports findings from other studies that identified these three variables confer higher risks of PTSD [26–29].

Having a physically injury was an influential risk factor in this study, which was consistent with several studies in this field [30, 31]. Disability is likely to reduce health related quality of life in these people, and might be conducive to PTSD. It is also possible that people who are injured in an earthquake witnessed the deaths of other people when the earthquake struck. The mud brick buildings were badly damaged in the earthquake, which caused a lot of casualties.

Our results represent that being trapped was an important factor that increased the likelihood of PTSD, similar to the findings of a previous study [32]. Undeniably, being trapped is an extremely traumatic experience. Thus, the persistence of PTSD symptoms in this group appears to be related to reminders such as rubble, destroyed buildings, and casualties. However, a study that was conducted by Kadak et al., did not find being trapped as a risk factor [33].

Our study illustrates that people who lost a relative or a friend were more likely to develop PTSD. Some PTSD studies after earthquakes showed the same relationship [34, 35]. It can be due to the fact that losing a family member removes some of the support resources from close relationships at a times of extreme need, increases the psychological stress, and makes people more susceptible to PTSD.

The negative relationship between having high educational degree and PTSD was found in the current study, which was similar to the findings of a former study [36]. High educated people were associated with a lower probability of being classified into the Moderate class compared to Low class. It is reasonable to say that, high level of education can indirectly affect economic resources, social status, social networks, and health behavior. However, our study indicates that there was no significant difference between education levels for comparing the Severe and Low classes. This supports the results of a prior study reported by Cofini et al. [37].

We find no associations between marital status and gender with PTSD. This is consistent with the findings of most other studies [38–40]. In contrast, a study reported an association with marital status and PTSD and identified that being unmarried conferred a higher risk of PTSD [41]. The effect of gender on PTSD following traumatic experiences has been documented by Zhou et al. and, Xu and Song also reported the positive relationship between being female and PTSD [42, 43]. This may be because over time, the severity effects of some risk factors may decrease and the pain may heal over time.

Significant association was not revealed between PTSD and loss of houses in our study, which was consistent with a previous study [44]. Nevertheless, Tian et al. and Yin et al. confirmed the role of loss and seriously damaged of houses after a disaster as a positive risk factor of PTSD [45, 46].

This study was performed a long time after the earthquake disaster with a large sample size and also, to the best of our knowledge, is the only study applying LCA in survivors of this disaster. The results of this study revealed that preparation and psychological interventions for PTSD are necessary for earthquake survivors. Psychiatric treatment programs for survivors, particularly the elderly, and those who have been injured, trapped or have lost family members or colleagues are essential, even after a long period of time. Earthquake survivors are usually forgotten after a while. This study showed that 17 years after the earthquake, survivors still need social support. Allocation of funds by the government for free counseling and the intermittent visits of these survivors by psychiatrists and psychologists, construction of recreational centers, sports and libraries to fill the survivors' leisure time seem necessary. Helping the survivors is expected to also improve the mental health of the next generation and their children, because the mental health of the children depends on that of their parents.

There are a number of limitations that should be considered when interpreting our findings. Bam’s adjacent towns and rural areas were not investigated, while many of them had been affected by the earthquake. Moreover, some people who had experienced the earthquake have immigrated from Bam city and, therefore, were not included in this study. This study was cross-sectional and a longitudinal study may provide more accurate results. In this study, the PCL-C was used for assessing the level of post-injury stress symptoms. The DSM-5 symptom system is currently used by the academic community and it differs from the PCL-C because the PCL-C is based on DSM-IV. DSM-5 needs negative alterations in cognitions and mood compared to DSM-IV.

In summary, the Bam earthquake was one of the most disastrous earthquakes of the last century in the world and the Middle East, and the results showed that the psychological follow-up of the survivors should continue to be one of the concerns of the health care system.

## Conclusions

In the event of an earthquake, efforts to rescue earthquake victims from the rubble must be carried out at high speed, as staying under the rubble and the wounds and injuries show the presence of relatively severe PTSD even after 17 years. We recommend that disaster mental health services should systematically screen and the afflicted survivors should not be forgotten. To overcome this problem, the earthquake survivors must be constantly engaged, and there must be recreational and welfare facilities, and the government must provide funding for free counseling for them.

## Data Availability

The datasets used and/or analyzed during the current study are available from the corresponding author on reasonable request.

## Abbreviations

PTSD: Post-traumatic stress disorder
LCA: Latent class analysis;
LCR analysis: Latent class regression analysis
PCL-C: PTSD Checklist–Civilian Version
DSM-IV: Diagnostic and Statistical Manual of Mental Disorders, Fourth Edition
BIC: Bayesian Information Criterion
AIC: Akaike information criterion
